# Role of Ultrasound Imaging in the Prediction of *TRIM67* in Brain Metastases From Breast Cancer

**DOI:** 10.3389/fneur.2022.889106

**Published:** 2022-06-20

**Authors:** Zhidong Xuan, Ting Ma, Yue Qin, Yajie Guo

**Affiliations:** Department of Ultrasound, Cangzhou Central Hospital, Cangzhou, China

**Keywords:** breast cancer, brain metastases, ultrasonography, TRIM67, biomarkers

## Abstract

**Objectives:**

Ultrasound (US) imaging is a relatively novel strategy to monitor the activity of the blood–brain barrier, which can facilitate the diagnosis and treatment of neurovascular-related metastatic tumors. The purpose of this study was to investigate the clinical significance of applying a combination of US imaging outcomes and the associated genes. This was performed to construct line drawings to facilitate the prediction of brain metastases arising from breast cancer.

**Methods:**

The RNA transcript data from The Cancer Genome Atlas (TCGA) database was obtained for breast cancer, and the differentially expressed genes (DEGs) associated with tumor and brain tumor metastases were identified. Subsequently, key genes associated with survival prognosis were subsequently identified from the DEGs.

**Results:**

Tripartite motif-containing protein 67 (*TRIM67*) was identified and the differential; in addition, the survival analyses of the TCGA database revealed that it was associated with brain tumor metastases and overall survival prognosis. Applying independent clinical cohort data, US-related features (microcalcification and lymph node metastasis) were associated with breast cancer tumor metastasis. Furthermore, ultrasonographic findings of microcalcifications showed correlations with *TRIM67* expression. The study results revealed that six variables [stage, *TRIM67*, tumor size, regional lymph node staging (N), age, and HER2 status] were suitable predictors of tumor metastasis by applying support vector machine–recursive feature elimination. Among these, US-predicted tumor size correlated with tumor size classification, whereas US-predicted lymph node metastasis correlated with tumor N classification. The *TRIM67* upregulation was accompanied by upregulation of the integrated breast cancer pathway; however, it leads to the downregulation of the miRNA targets in ECM and membrane receptors and the miRNAs involved in DNA damage response pathways.

**Conclusions:**

The *TRIM67* is a risk factor associated with brain metastases from breast cancer and it is considered a prognostic survival factor. The nomogram constructed from six variables—stage, *TRIM67*, tumor size, N, age, HER2 status—is an appropriate predictor to estimate the occurrence of breast cancer metastasis.

## Introduction

Brain metastases (BM) have gradually become the most common malignancy of the central nervous system, with 20–40% of patients with cancer developing BM (1, 2). Primarily, BM arises from tumors in the lungs and the breast; however, they are observed at other sites as well (3). Annually, the global incidence of BM has progressively increased and is associated with a poor prognosis. The overall survival varies from 3 to 25 months depending on tumor type and subtype, BM has become a clinically important disease that poses a remarkable threat to human health (2, 4). An estimated 30–50% of patients with metastatic breast cancer (MBC) develop BM (5, 6). The development of BM depends on the action of neurovascular units (NVU), thereby emphasizing the unique and close relationship between the vessels and cells in the brain (7). Brain endothelial cells (BECs) occupy a central position in the NVU and they are regulated by neuronal cells in the formation and maintenance of the blood–brain barrier. Tumor cells must overcome the tightly connected endothelial barrier of BECs to ensure the occurrence of BM (8). Breast cancer (BRCA) is a relatively common type of tumor that leads to the formation of neurovascular metastases and has a very poor prognosis; furthermore, its presence is a notable health risk for patients with cancer (9, 10). Identifying neurovascular metastases from brain tumors is essential for monitoring the prognosis of patients with breast cancer.

Understanding ultrasound (US) signals related to neurovascular tissues in disease conditions will be instrumental in elucidating disease mechanisms and identifying potential diagnostic and treatment targets (11). Ultrasound examinations expose the patient to considerably low levels of radiation, and the test itself is non-invasive and reproducible. Considering this, US examinations are a common and convenient modality in the management of breast cancer (12). Previously, several studies have examined the role of US imaging in the identification of breast cancer metastasis and prognosis. Ultrasound imaging has a suitable degree of sensitivity and specificity in diagnosing breast cancer metastasis to axillary lymph nodes (13). Tumors diagnosed *via* breast US have better prognostic features in terms of size and lymphovascular invasion (14). Furthermore, the formation of microcalcifications affected the growth and infiltration of breast cancer and is associated with disease prognosis in patients (15). Ultrasonography can provide a multidirectional, multi-angle sweep of microcalcified breast cancer lesions; this feature is more widely used in the screening and diagnosis of microcalcifications in breast cancer (16). In addition, ultrasonographic observation of lesion blood flow grading correlated with positive ER expression of the estrogen receptor, which can indirectly indicate the sensitivity of breast cancer to treatment (17, 18). However, neurovascular metastases in breast cancer were closely related to gene expression characteristics, and the items examined *via* breast US imaging alone were biased as they do not adequately reflect this.

The recent developments in bioinformatics have facilitated the diagnosis and treatment of the disease with the help of large-scale biological data (19–22). The considerable amount of RNA sequencing data and the increasing number of prognostic factors and therapeutic targets identified have considerably aided in the examination of the mechanisms related to cancer development and clinical treatment (23, 24). One study used RNA sequencing to probe the cellular origin and evolution of breast cancer in *BRCA1* mutant carriers (25). Additionally, bioinformatics has reportedly been used to identify differentially expressed genes (DEGs) associated with breast cancer metastasis and provide potential therapeutic targets for metastatic breast cancer (26). Bioinformatics data combined with imaging findings can also play a crucial role in breast cancer diagnosis and prognosis. Breast US imaging combined with plasma miR-21 and miR-27a significantly improves the diagnostic efficiency of breast cancer, thereby facilitating early clinical intervention (27). We believe that a combination of US imaging and transcriptional profiling can predict the risk of cerebral neurovascular metastases in patients with breast cancer.

In this study, we aimed to investigate the genes associated with the development of neurovascular BM in patients with breast cancer and to explore the role of ultrasonography in the examination of tumor metastases. Furthermore, this was performed to identify potential diagnostic and therapeutic targets/features to monitor and improve the risk of BM in patients with breast cancer and to extend their life expectancy.

## Methods

### Clinical Information and TCGA Data Acquisition

We retrospectively analyzed patients with surgically confirmed breast cancer who presented themselves to our hospital between August 2015 and March 2021. The following cases were excluded in this study: (1) cases of male breast cancer; (2) cases where the elapsed time between surgery and US was more than 2 weeks; (3) cases with incomplete clinical information; (4) cases with unilateral multiple foci; and (5) cases with primary malignancy at a different site. Using the above exclusion criteria, a total of 97 breast cancer patients with adequate follow-up were finally screened. Postoperative pathological staging was based on the American Joint Committee on Cancer (AJCC) TNM staging criteria for breast cancer. Informed consent was obtained from all patients. Consent was also obtained from the Ethics Committee of Cangzhou Central Hospital. The Cancer Genome Atlas (TCGA; https://portal.gdc.cancer.gov/) project database was searched for 1,041 breast invasive carcinoma (BRCA) patient samples consisting of 60 tumors with non-brain metastasis (nBM–BRCA), 4 tumors with brain metastasis (BM–BRCA), and 977 tumors without distant brain metastasis (nDM–BRCA). Transcriptome data were downloaded in transcripts per million (TPM) unit format and were log-transformed.

### Tumor Features Evaluation by Ultrasonography

Sonography was performed using a Siemens-2000 color Doppler diagnostic US machine with a probe frequency of 7–15 Hz. Different clinical features were analyzed using US including tumor size, microcalcification status, blood flow grading, and lymph node metastases. The longest measured diameter of a lesion in any section scan was considered as the size of the primary tumor. The size (<1-mm in diameter), number, and distribution of calcified spots were used as bases to determine the status of microcalcifications in breast cancer. Blood flow was graded into four levels according to Adler blood flow grading: Grade 0, no blood flow in the tumor lesion; Grade I, little blood flow, i.e., one or two spots of blood flow (<1-mm in diameter) are visible; Grade II, moderate blood flow, i.e., one main vessel or several small vessels visible; Grade III, significant blood flow, i.e., four or more vessels visible (28). Ultrasound was used to assess axillary lymph node metastases by (1) high-frequency US sonograph showing a lymph node thickness of more than 3 mm or a solid axillary hypoechogenicity, or (2) color flow imaging showing abundant blood flow within the lymph nodes.

### Logistic Regression Analysis

To explore the relationship between tumor features and metastasis, logistics regression was used to determine the predictive power of the observed tumor features from the sonographs in relation to tumor metastasis (29, 30). The purpose of using logistic regression in this step is not to train diagnostic classifiers, but to test for multivariate differences. First, univariate logistics regression was used to analyze the general clinical and US-related features of the patients, with the relevant characteristics screened at *p* < 0.05 level of significance. Then, the screened US and clinical characteristics were subjected to multivariate logistic regression analysis. The odds ratio (OR) and 95% confidence interval (95% CI) for each variable were assessed as statistical indicators. The variables with a significance level of *p* < 0.05 in both univariate and multifactorial logistic regression analyses were considered as independent predictors of tumor metastasis.

### Bioinformatics Analysis

Distant metastases- and BM-associated differentially expressed genes (DEGs) were identified through the differential analysis of transcriptomic data from tumor tissue samples using the limma package (31). The DEGs were filtered based on a threshold value of *p* < 0.05. Subsequently, intersection analysis of DM- and BM-associated DEGs was performed to identify genes that were differentially expressed in both DM and BM. From the obtained gene list, survival analysis was employed to further screen for prognosis-related genes.

### Quantitative Reverse Transcription-PCR

Quantitative RT-PCR (qRT-PCR) was used to quantify the RNA expression levels of key genes present in tumor samples collected after surgical resection. The total RNA was extracted using the mirVana™ miRNA isolation kit (Ambion) according to the manufacturer's protocol. The harvested total RNA was reverse transcribed to cDNA using RevertAid first strand cDNA synthesis kit (Thermo Scientific™, USA). The qRT-PCR was performed using SYBR® Green Realtime PCR master mix (Toyobo, Japan) on ABI 7500 real-time PCR system (Applied Biosystems, CA, USA). Using GAPDH as the reference gene, the 2^−Δ*ΔCT*^ method was used for the relative quantification of gene expression levels. In the final conclusion, gene expression levels were reported as the fold-change (FC) relative to the reference.

### Support Vector Machine–Recursive Feature Elimination

To screen for features associated with tumor metastases, an exploratory analysis of the patient's general characteristics, US-related features, postoperative pathological features and key genes was applied support vector machine–recursive feature elimination (SVM–RFE) (32). In supervised machine learning, support vector machines (SVMs) are often used for non-linear classification. In addition, SVM showed good performance in processing data sets with small sample sizes (30, 33, 34). Support vector machine–recursive feature elimination filtered the variables of the prediction results by iteratively removing redundant feature variables. Using a cross-validation approach, the root mean squared error (RMSE) parameter was used to assess the predictive performance of the model and to screen for the best combination of variables. The entire dataset was divided into a training set and a test set in a ratio of 7:3. The training set was used for feature selection and model training, and the test set was used for testing the model. The area under curve (AUC) of the receiver operating characteristic curve (ROC) was also applied to assess the predictive performance of the model; pROC curves are plotted using the “pROC” package.

### Nomogram Construction

A principal component analysis (PCA) was applied to further extract features from the SVM–RFE that reflect tumor metastasis in order to determine whether they provided adequate information for classification. To develop predictive models for these characteristics, multivariate logistic regression was applied. Decision curve analysis (DCA) assesses the usefulness and safety of clinical prediction models by calculating the net benefit ratio for different threshold probabilities. To facilitate clinical use, a visualization tool was constructed by applying the “rms” to the nomogram of this clinical prediction model. Calibration curves are used to assess the agreement between predicted probabilities and actual outcomes.

### Survival Analysis

For BRCA patients, Kaplan–Meier (KM) curves were used to calculate the probability of overall survival (OS) at 1, 3, and 5 years. Cox regression analysis was used to perform a difference-in-difference test analysis of the survival curves between the two groups. The time-dependent ROC and area under the curve (AUC) values were used to quantify the predictive performance of the target gene for survival in BRCA patients (35). Survival data were analyzed using “survival” package, and visualization was carried out using survminer R package.

### Gene Set Enrichment Analysis

Analysis of functional pathways dysregulated in TCGA–BRCA tumor samples with dysregulated genes using Gene Set Enrichment Analysis (GSEA) as the previous researches (36–39). The pathways in the analysis were annotated using the “c2.cp.v7.2.symbols.gmt [Curated]” gene set from MSigDB Collections (https://www.gsea-msigdb.org/gsea/msigdb/). Package clusterProfiler was used to calculate the statistical significance of the enrichment of functional pathways (40). The threshold of statistical significance for functional pathway enrichment was set at adjusted *p* < 0.05.

### Statistical Analysis

All statistical analyses and figures were obtained using the R (version 4.0.2) software. In the statistical analysis of patients' baseline data, the Pearson's Chi-squared test or Fisher's exact test was used to test the count data. Differences in the measurements between the two groups were determined using the Wilcoxon rank–sum test or unpaired *t*-test. Correlation analysis was performed using the Spearman method. The results from these statistical analyses were plotted using the “ggplot2” package. Computed *p*-values of <0.05 were considered to be statistically significant.

## Results

### Baseline Patients' Information

Among the 97 patients with breast cancer enrolled in the clinical cohort study, distant metastases (DM) developed and did not develop in 34 and 63, respectively. The clinical baseline information, postoperative pathological features, and US features of the patients with breast cancer are summarized in Table 1. Patients with breast cancer with and without DM showed significant differences in terms of TNM stage, metastasized staging (M), tumor size staging (T), regional lymph node staging (N), HER2, tumor size, microcalcification, Adler blood flow grading, and prediction of lymph node metastasis. No statistically significant differences were observed in terms of age, menopausal status, tumor location, histology, ER, and PR. However, microcalcifications confirmed by preoperative US, higher blood flow grading, and lymph node metastases may indicate a higher risk of DM.

**Table 1:** Baseline characteristics of patients in the clinical cohort.

**Characteristic**	**Metastasis**		
	**Yes**	**No**	***P***
*N*	34	63	
Age, mean ±*SD*	59.29 ± 12.38	55.87 ± 11.86	0.185
Menopause, *n* (%)			0.413
Premenopause	9 (9.3%)	18 (18.6%)	
Perimenopause	5 (5.2%)	4 (4.1%)	
Postmenopause	20 (20.6%)	41 (42.3%)	
Location, *n* (%)			0.756
Left	17 (17.5%)	35 (36.1%)	
Right	17 (17.5%)	28 (28.9%)	
Histology, *n* (%)			0.712
Infiltrating ductal carcinoma	20 (20.6%)	41 (42.3%)	
Infiltrating lobular carcinoma	8 (8.2%)	14 (14.4%)	
Others	6 (6.2%)	8 (8.2%)	
Stage, *n* (%)			<0.001**
Stage I	0 (0%)	11 (11.3%)	
Stage II	6 (6.2%)	41 (42.3%)	
Stage III	20 (20.6%)	11 (11.3%)	
Stage IV	8 (8.2%)	0 (0%)	
M, *n* (%)			<0.001**
M0	26 (26.8%)	63 (64.9%)	
M1	8 (8.2%)	0 (0%)	
*N, n* (%)			<0.001**
N0	1 (1%)	30 (30.9%)	
N1	15 (15.5%)	23 (23.7%)	
N2	10 (10.3%)	7 (7.2%)	
N3	8 (8.2%)	3 (3.1%)	
T, *n* (%)			0.007**
T1	3 (3.1%)	19 (19.6%)	
T2	19 (19.6%)	37 (38.1%)	
T3	10 (10.3%)	6 (6.2%)	
T4	2 (2.1%)	1 (1%)	
ER, *n* (%)			0.592
Negative	10 (10.3%)	14 (14.4%)	
Positive	24 (24.7%)	49 (50.5%)	
PR, *n* (%)			0.166
Negative	17 (17.5%)	21 (21.6%)	
Positive	17 (17.5%)	42 (43.3%)	
HER2, *n* (%)			<0.001**
Negative	17 (17.5%)	53 (54.6%)	
Positive	17 (17.5%)	10 (10.3%)	
*TRIM67*, median (IQR)	−0.08 (−0.52, 0)	−0.41 (−0.94, −0.02)	0.021*
US examination			
Size/mm, median (IQR)	33 (24.12, 53.88)	27.3 (17.7, 38.4)	0.013*
Microcalcification, *n* (%)			<0.001**
No	5 (5.2%)	35 (36.1%)	
Yes	29 (29.9%)	28 (28.9%)	
Adler blood flow grading, *n* (%)			0.005**
0–I	9 (9.3%)	37 (38.1%)	
II–III	25 (25.8%)	26 (26.8%)	
Lymph node metastasis, *n* (%)			<0.001**
No	3 (3.1%)	29 (29.9%)	
Yes	31 (32%)	34 (35.1%)	

*Ultrasound examination include tumor size calculation, microcalcification, altered blood grading, and lymph node metastasis prediction*.

*p < 0.05, **p < 0.01.

### Ultrasound Predicts BRCA Metastasis

Typically, the logistics model is used to screen for risk factors associated with the poor outcomes. In this section, we have included only general clinical features and ultrasonography-related features for analysis to explore the ability of preoperative ultrasonography and diagnosis to assess the likelihood of DM in patients with tumors. Results of logistic regression analysis to screen the predictors associated with DM are presented in Table 2. Univariate logistic analysis showed that tumor size in US imaging (*p* = 0.015), microcalcification (*p* < 0.001), Adler blood flow grading (*p* = 0.003), and lymph node metastasis (*p* = 0.001) were significantly associated with the occurrence of DM in patients with breast cancer. Further multivariate logistic regression analysis showed that microcalcification (*p* = 0.02) and lymph node metastasis (*p* = 0.011) were independent predictors of DM.

**Table 2:** Univariate and multivariate logistics regression analysis with US-related variables.

**Variables**	**Univariate logistic regression**			**Multivariate logistic regression**		
	**OR**	**95% CI**	***p***	**OR**	**95% CI**	***p***
Age^#^	1.02	0.99, 1.06	0.185			
**Premenopause**			
Perimenopause	0.39	0.09, 1.63	0.194			
Postmenopause	0.4	0.08, 1.86	0.243			
**Location (Left)**			
Right	1.25	0.54, 2.9	0.601			
Size^#^	1.04	1.01, 1.07	0.015*	1.02	0.98, 1.05	0.366
**Microcalcification (No)**			
Yes	7.25	2.66, 23.51	<0.001**	5.1	1.38, 22.33	0.02*
**Adler blood flow grading (I–II)**			
II–III	3.95	1.63, 10.26	0.003**	0.97	0.25, 3.39	0.957
**Lymph node metastasis (No)**			
Yes	8.81	2.78, 39.39	0.001**	6.19	1.71, 30.79	0.011*

# *Continuous variable; CI, confidence interval*.

*p < 0.05, **p < 0.01.

### The *TRIM67* Expression Correlates With BM and DM

**Figure 1:**
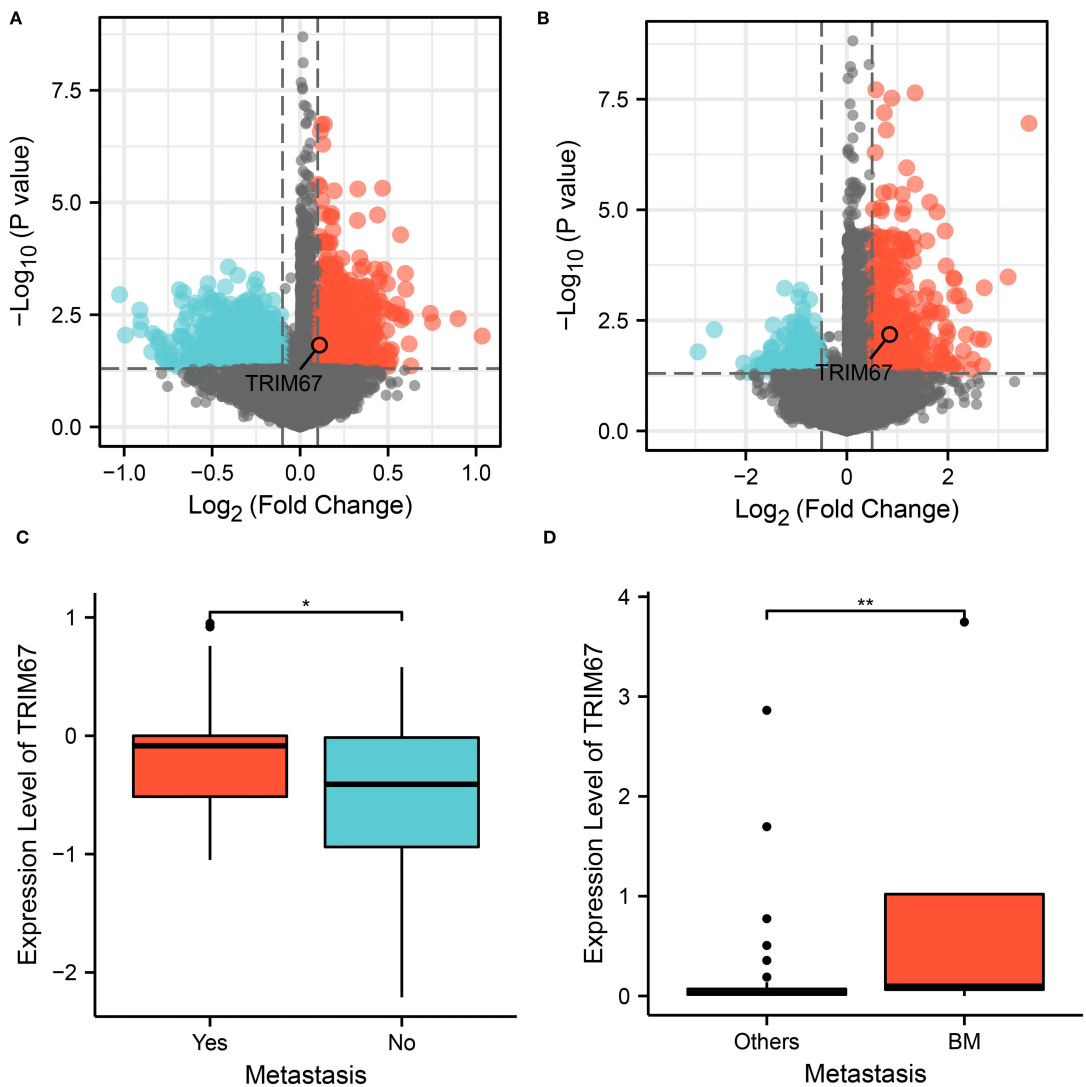
Analysis of variance showed that *TRIM67* was upregulated in both DM and BM. **(A)** Volcano plot showing differentially expressed genes between the DM and nDM groups, where *TRIM67* was found to be upregulated in DM; **(B)** Differential expression analysis between BM and nBM groups showed that *TRIM67* was upregulated in BM; **(C)** There was a statistically significant difference in *TRIM67* expression between the DM and nDM groups (*p* = 0.021); **(D)** There was a statistically significant difference in *TRIM67* expression between the BM and nBM groups (*p* = 0.007).

We performed differential expression analysis of the RNA transcript data between the two groups at the tumor tissue level. Differential analysis of The Cancer Genome Atlas (TCGA) data and patients with breast cancer with and without DM revealed 3,759 DEGs, which were identified as DM-associated DEGs (Figure 1A). Similarly, 2,029 BM-associated DEGs were identified between the BM and nBM groups (Figure 1B), and Cox analysis revealed 1,727 survival-associated DEGs. Cross-tabulation analysis subsequently showed that four genes—*TRIM67*, SFXN2, CEL, and IGKV6-21—were associated with DM, BM, and survival outcomes. Additionally, *TRIM67* expression was significantly different between the DM and nDM groups (*p* = 0.021) and between the BM and nBM groups (*p* = 0.007) (Figures 1C,D). Therefore, *TRIM67* was used for further analysis.

### Ultrasonography and *TRIM67* Expression

**Figure 2:**
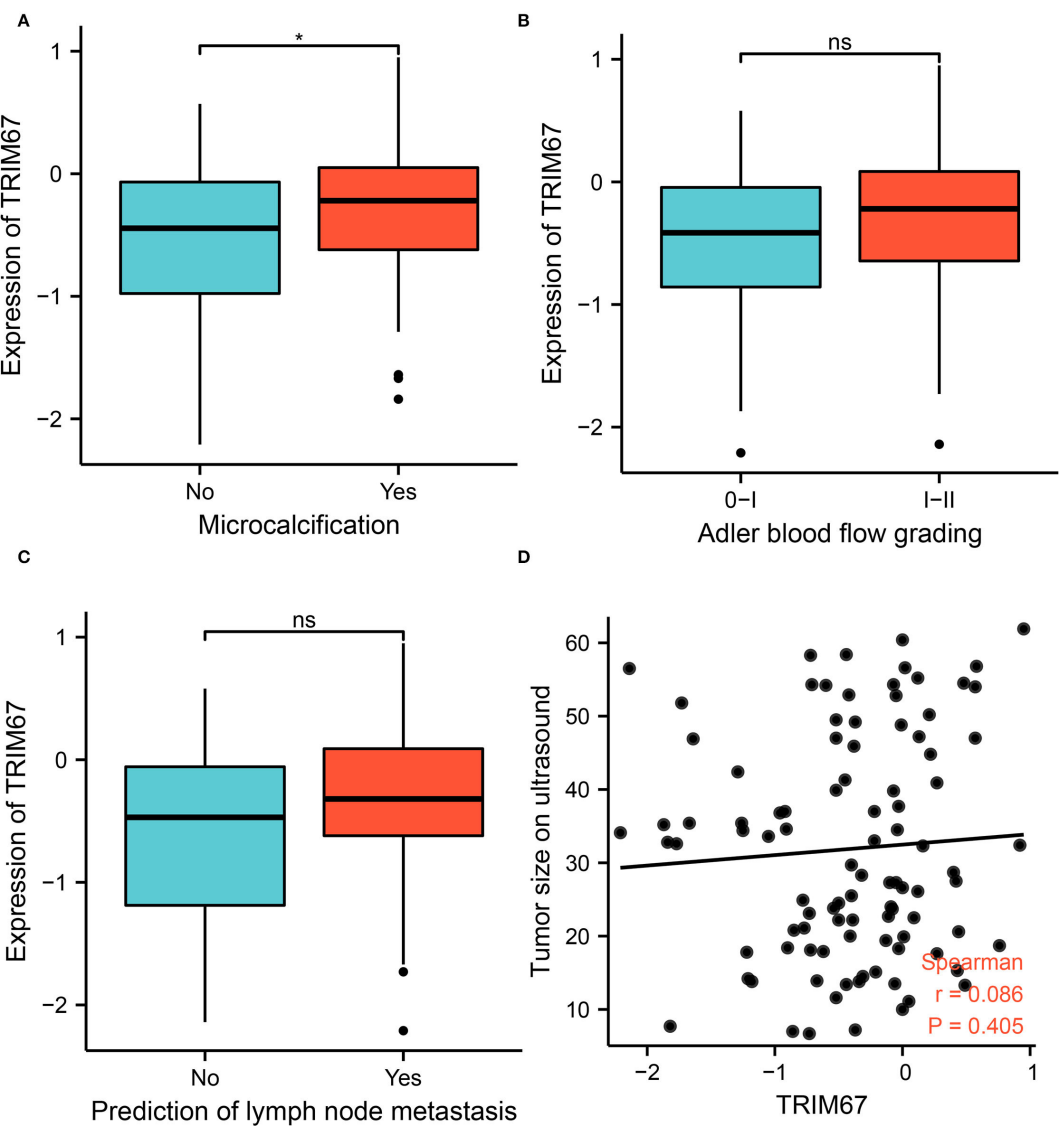
Relationship between US-related features and *TRIM67* expression. **(A)** Microcalcifications in tumor tissue correlated with *TRIM67* expression (*p* = 0.027); **(B,C)** The bar graph shows the relationship between Adler blood flow grading and lymph node metastasis in relation to differential expression of *TRIM67*; **(D)** Correlation analysis of tumor size on US and *TRIM67* expression.

To investigate whether US can assess *TRIM67* expression, an exploratory analysis of the relationship between US findings and *TRIM67* was performed. The results showed a significant difference between microcalcifications and *TRIM67* expression in the US findings (*p* = 0.027). The *TRIM67* expression was higher in the group with microcalcifications than in the group without microcalcifications (Figure 2A). the *TRIM67* expression showed no significant differences from Adlerian blood flow grading, lymph node metastasis prediction, and tumor size (Figures 2B–D). However, the results appeared to imply that a higher blood flow grading, positive lymph node metastasis prediction, and larger tumor size correlated with a higher *TRIM67* expression.

### The SVM–RFE Shows Variables Associated With Prognosis

**Figure 3:**
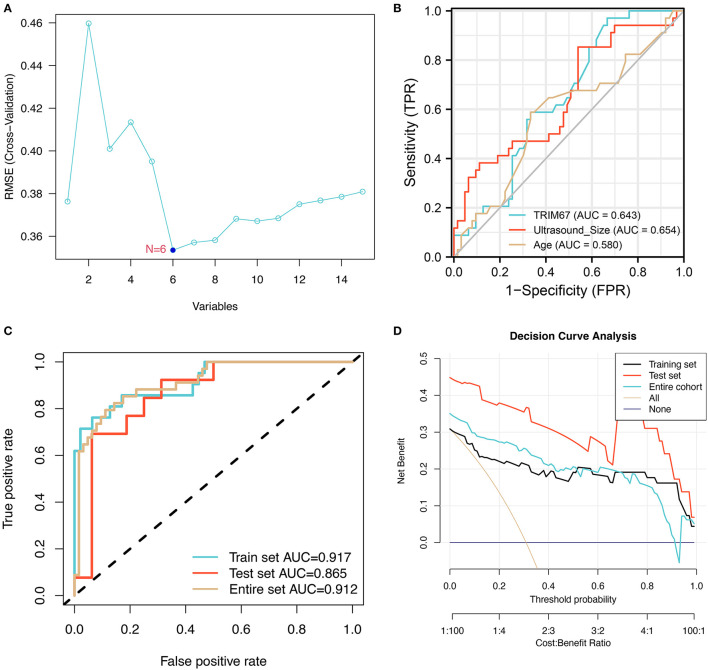
The training process and performance evaluation of SVM-RFE models. **(A)** The variable screening process showed the smallest RMSE for the model when six variables were selected; **(B)** The ROCs show the predictive power of *TRIM67*, tumor size, and age on tumor metastasis in a selection of six variables; **(C)** The ROC shows the predictive power of a logistic regression model combining six indicators for tumor metastasis; **(D)** The DCA suggested that the clinical prediction model has a good range of clinical use in the training, test, and overall groups.

The general patient characteristics, the ultrasonographic information, the postoperative pathological information, and the predictive power of *TRIM67* in tumor metastasis were assessed using SVM–RFE. The results showed that the model had the lowest RMSE (Figure 3A) when six variables (stage, *TRIM67*, tumor size, N, age, and HER2 status) were included in the SVM model. The receiver operating characteristic (ROC) curves of *TRIM67* [area under ROC curve (AUC), 0.643], tumor size (AUC, 0.654), and age (AUC, 0.580) for the prediction of tumor metastasis are shown in Figure 3B, indicating their general predictive ability. The AUCs of the multifactor logistic regression model constructed by combining the above six characteristics were 0.917, 0.865, and 0.912 for the training group, test group, and all groups, respectively (Figure 3C). The clinical prediction model showed an acceptable predictive power for tumor metastasis. Table 3 shows the coefficients for these six variables in the clinical prediction model. A positive correlation was found between the probability of tumor metastasis and stages III–IV, *TRIM67*, size, N1–N3, and positive HER2. The DCA of the clinical prediction model in the training, test, and overall groups is shown in Figure 3D, demonstrating the large clinical range of the model.

**Table 3:** Included variables of the nomogram-based model.

**Variables**	**β**	**Odds ratio (95% CI)**	***p***
(Intercept)	−4.220	0.01 (0–0.15)	0.003**
Stage (III–IV vs. I–II)	2.380	10.81 (2.98–47.76)	0.001**
*TRIM67*	1.240	3.46 (1.13–13.77)	0.047*
Size	0.020	1.02 (0.97–1.07)	0.478
Age (>50 vs. <50, years)	0.240	1.28 (0.33–5.08)	0.721
N (N1–3 vs. N0)	1.910	6.76 (0.96–138.87)	0.098
HER2 (Positive vs. Negative)	1.530	4.62 (1.27–19.14)	0.025*

*β, the regression coefficient; CI, confidence interval*.

*p < 0.05, **p < 0.01.

### Construction of a Clinical Prediction Model for Tumor Metastasis

**Figure 4:**
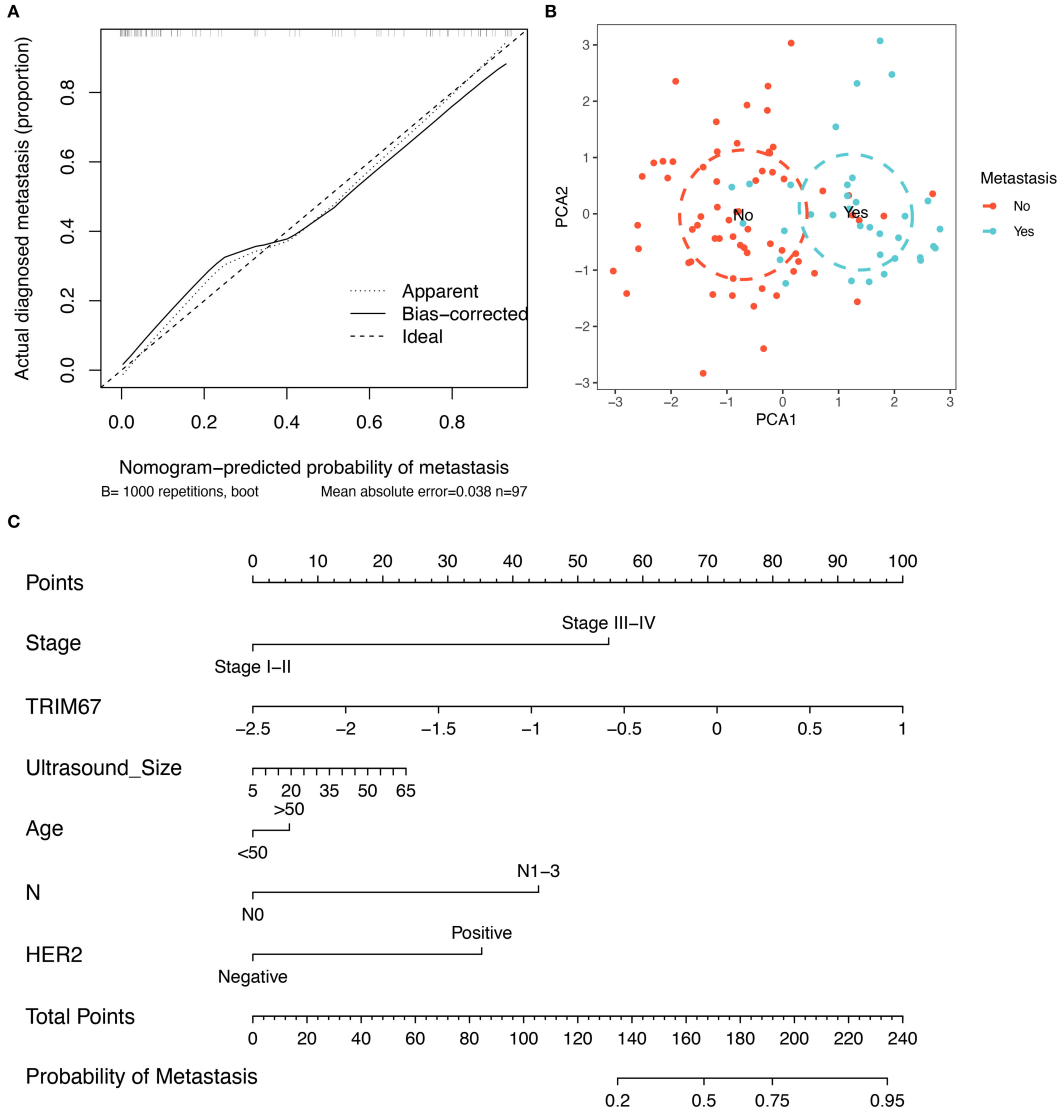
Construction of a clinical prediction model for tumor metastasis. **(A)** The correction curves show that the predicted outcomes of this clinical prediction model are in good agreement with the true outcomes of the patients; **(B)** The PCA visualization demonstrates the efficacy of using the six variables to differentiate patients with breast cancer who developed tumor metastases; **(C)** The nomogram, constructed based on a clinical prediction model, allows an assessment of the likelihood of tumor metastasis based on these six variables.

Developing a predictive model has the potential to be useful in clinical practice is our aim. First, the correction curves showed a good degree of agreement between the predicted outcomes of this constructed clinical prediction model and the patients' perceptions of the true outcomes (Figure 4A). Subsequently, the PCA analysis showed that patients with breast cancer with and without tumor metastases could be differentiated according to the variables used in the model (Figure 4B). Ultimately, based on a multivariate logistic regression model, the nomogram was prepared to facilitate its use in clinical settings (Figure 4C). Nomograms are composed of selected feature variables and their corresponding scores. The total score was obtained by summing the corresponding scores for each variable before converting it directly to obtain the probability of tumor metastasis. Therefore, the nomogram ensures that a patient's likelihood of developing metastases can easily be assessed by using important clinical features, preoperative US findings, and postoperative pathological information.

### Relationship Between *TRIM67* and US and Postoperative Pathology

**Figure 5:**
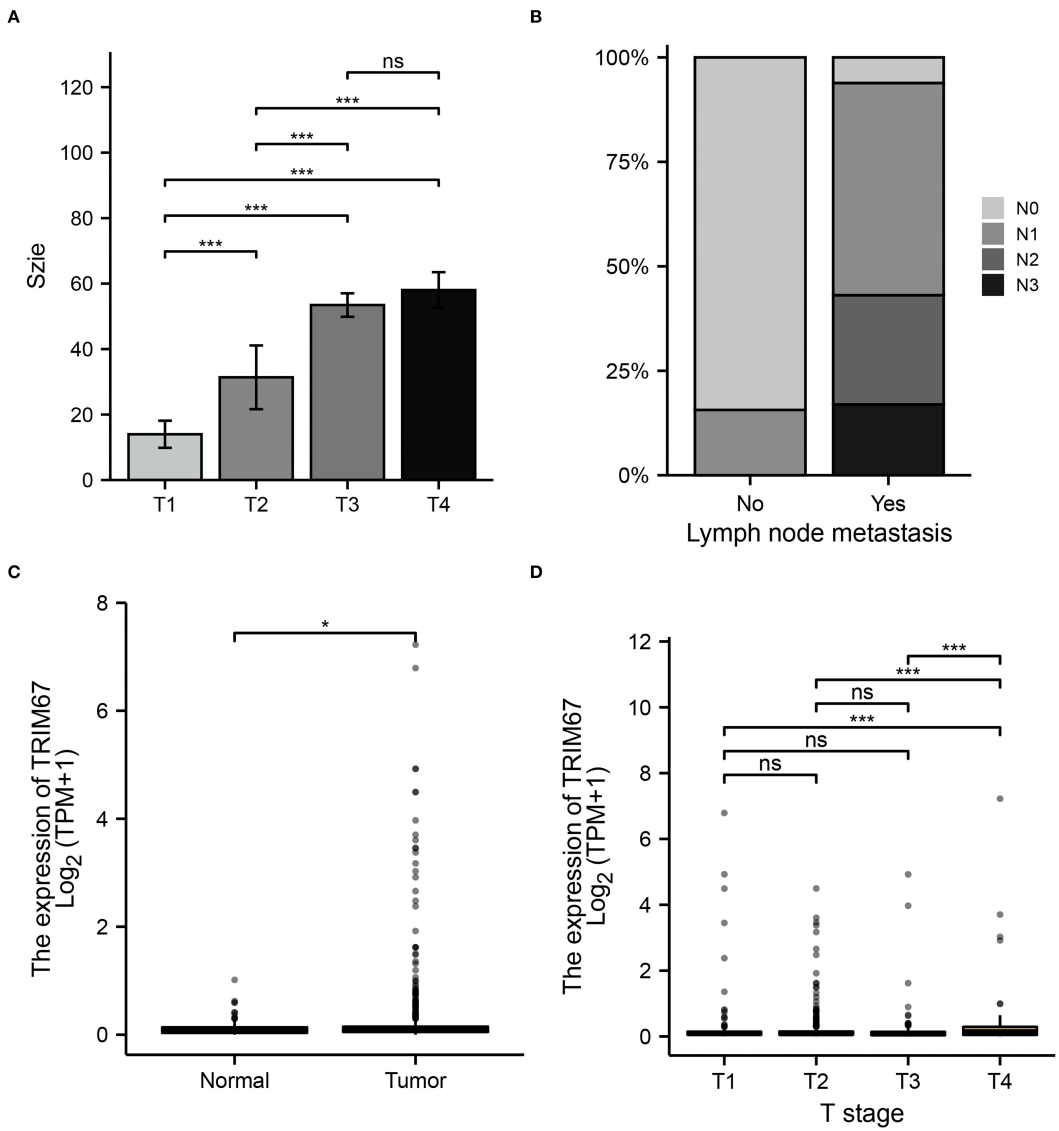
Relationship between preoperative ultrasonography and *TRIM67* and postoperative pathological features. **(A)** The bar chart shows a correlation between the T-grading of the tumor and the size of the tumor as measured using preoperative US; **(B)** There was a correlation between US-predicted lymph node metastasis and the N grade of the tumor; **(C)** There were differences in *TRIM67* expression between normal and tumor tissues; **(D)** There were differences in *TRIM67* expression between different tumor T-grades.

Variables in the clinical prediction model have been discussed further in this section. The tumor size observed on US examinations appeared to correlate with the T stage in the TNM classification (Figure 5A). Furthermore, the distribution of N grades in the TNM grading showed significant differences between the US-predicted lymph node metastasis group and the US-predicted non-lymphoid node metastasis group (*p* < 0.05, Figure 5B). These results demonstrated that, in the clinical prediction model, the tumor size measured using US reflects the T-staging of the tumor, whereas the N-staging corresponds to the lymph node metastasis predicted by US. This illustrated the important role of ultrasonography in predicting the development of tumor metastases in patients with breast cancer. In addition, normal and tumor tissues showed different *TRIM67* expression levels (Figure 5C). Additionally, significant differences in *TRIM67* expression were identified between T1, T2, T3, and T4 (Figure 5D). Therefore, T staging can reflect *TRIM67* expression to some extent. The abovementioned results demonstrated the complex interrelationship between ultrasonography-related features, postoperative pathological grading, and *TRIM67* expression.

### Elevated *TRIM67* Suggests a Poor Prognosis

**Figure 6:**
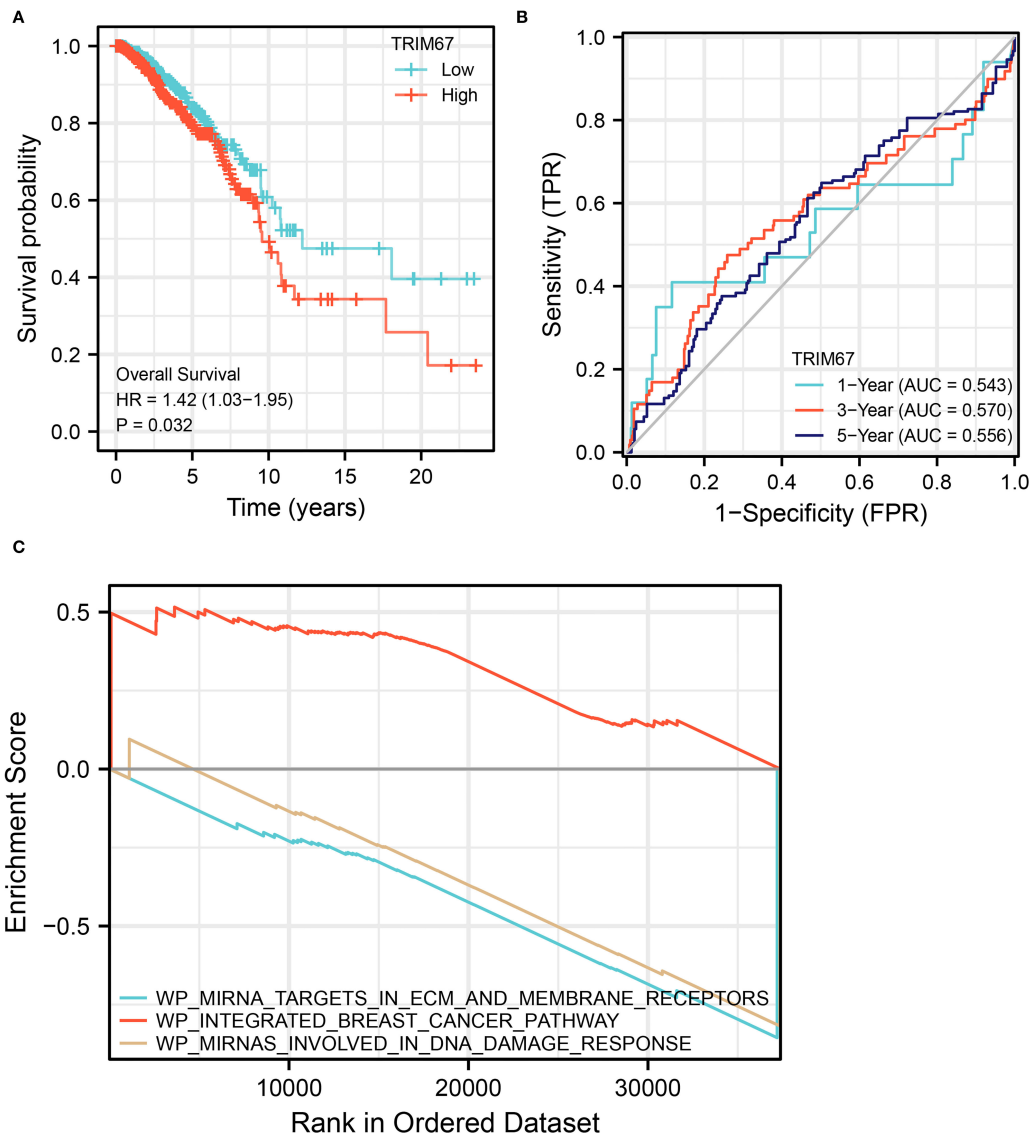
Survival analysis and GSEA for *TRIM67*. **(A)** KM curves showed significant differences (*p* = 0.032) in survival curves between high and low *TRIM67* groups for patients with breast cancer; **(B)** ROC shows the predictive power of *TRIM67* for overall survival at 1, 3, and 5 years; **(C)** GSEA shows the functional pathways that are enriched when *TRIM67* is upregulated.

The KM curve for *TRIM67* is shown in Figure 6A. Median overall survival time from the initial diagnosis of breast cancer was significantly shorter in the high *TRIM67* group than in the low *TRIM67* group (9.6 vs. 12.2 years, respectively, *p* = 0.031). Univariate Cox analysis showed a statistical difference between *TRIM67* expression and survival outcome (*p* = 0.032, HR = 1.42, 95% CI: 1.03–1.95). The ROC curves showed that the predicted AUC of *TRIM67* for overall survival at 1, 3, and 5 years were 0.543, 0.570, and 0.556, respectively (Figure 6B). These results suggested that elevated *TRIM67* was associated with a poor prognosis in patients with breast cancer.

### Pathways Enriched by Concomitant *TRIM67* Upregulation in BM

To further explore the possible functional pathways involved in *TRIM67* in BM in patients with breast cancer, we used RNA sequencing data from patients with breast cancer who developed distant tumor metastasis in TCGA for Gene Set Enrichment Analysis (GSEA). The results showed that in *TRIM67*-upregulated tumors, the integrated breast cancer pathway was upregulated, whereas the miRNA targets in ECM and membrane receptors and miRNAs involved in DNA damage response pathways were downregulated (Figure 6C). The *TRIM67* upregulation appeared to accompany the upregulation of the pathways related to breast cancer. In addition, *TRIM67* upregulation may be associated with the downregulation of certain miRNA-regulated functional pathways, including the role of membrane receptors targeted by miRNAs and their response in the DNA damage response. Alterations in these pathways may be associated with the development of BM in patients with breast cancer, and ultimately affect patient OS.

## Discussion

Ultrasound imaging is a new strategy for monitoring the activity of the blood–brain barrier, which can facilitate the diagnosis and treatment of metastatic tumors related to neurovascular tissues. In this study, we explored the possible role of US as well as *TRIM67* in the development of BM in breast cancer through a retrospective clinical cohort study and bioinformatics analysis. The *TRIM67* was identified as a factor associated with neurovascular metastasis, tumor metastasis, and prognostic survival in brain tumors in bioinformatics analysis of TCGA and breast cancer.

The *TRIM67* expression was correlated with microcalcifications on ultrasonography. Following this, we incorporated TRIM67 expression data, general clinical information, postoperative pathology, and US-related characteristics and screened six variables (stage, *TRIM67*, tumor size, N, age, and HER2 status) with the SVM–RFE method, which could predict the likelihood of tumor metastasis. Of these six features, tumor size measured using US correlates with the T classification of the tumor, whereas lymph node metastasis was predicted by US correlated with the N classification.

In this study, *TRIM67* was upregulated in patients with BM from breast cancer and DM. The previous studies on *TRIM67* in tumors have demonstrated that the *TRIM67* protein functions differently in different tumors. The *TRIM67* was reportedly downregulated in colorectal cancer and *TRIM67* inhibited tumor proliferation and metastasis (41, 42). The *TRIM67* promoted the progression of non-small cell lung cancer through the Notch pathway, including promoting cell proliferation, migration, and EMT (43). However, upregulation of *TRIM67* expression increased apoptosis in non-small cell lung cancer cells (44). Although the relationship between *TRIM67* and tumors remains unclear, all studies mentioned above reported that *TRIM67* was associated with tumor size, lymph node metastasis, tumor stage, and disease prognosis (41–43). Similar to the previous studies, we observed that *TRIM67* was associated with BM in breast cancer and was a risk factor for a poor prognosis. Microcalcifications on US findings correlated with *TRIM67* expression, and a higher blood flow grade, positive prediction of lymph node metastasis, and larger tumor size corresponded with higher *TRIM67* expression. In *TRIM67* upregulated tumors, the integrated breast cancer pathway was upregulated, whereas miRNA targets in ECM and membrane receptors and the miRNAs involved in DNA damage response pathways were downregulated. Therefore, these pathways may play an important role in the metastasis of breast cancer.

Microcalcification is an important early sign of breast cancer and its early diagnosis depends on the identification of suspected microcalcifications (45–47). The morphology and distribution of microcalcifications can be used to differentiate between benign and malignant lesions (48, 49). The previous studies have observed the presence of an association between microcalcifications and lymph node metastasis and prognosis in breast cancer (50, 51). BMP-2 is closely associated with microcalcification (50). The BMP signaling affects the invasiveness of breast cancer tumor cells (52). Similar to the previous studies, we observed that microcalcification and lymph node metastasis measured using US examinations were independent risk factors for distant tumor metastasis based on preoperative US. Ultrasound has been widely used to detect lymph node metastases in breast cancer. Ultrasound and needle biopsy of suspected lymph nodes could effectively identify the presence of lymph node metastases from breast cancer (53). Therefore, it is our understanding that microcalcification and lymph node metastasis play an important role in predicting breast cancer metastasis. In this study, *TRIM67* was associated with tumor microcalcification, and ultrasonography may reflect changes in *TRIM67* to some extent by monitoring the status of microcalcifications. The *TRIM67* expression is associated with DM in breast cancer; therefore, US combined with examination of *TRIM67* expression may have a better predictive function in breast cancer metastases.

The six variables screened by SVM–RFE were used to construct clinical prediction models. Using the nomogram, we can easily assess the likelihood of tumor metastasis in patients with breast cancer using important clinical features, preoperative US findings, and postoperative pathological information. Tumor size measured *via* US examination reflects the T-grade of the tumor, whereas the N-grade corresponds to the lymph node metastases predicted by US. Similar findings have been observed in the previous studies on the relationship between US and clinicopathological features of breast cancer. Li et al. reported that the pathological grading of breast cancer correlated with the shape of the tumor measured using US (54). Lamb et al. found that the grade and infiltration of breast cancer correlated with the echogenicity and tumor margin measured by US (55). This highlighted the important role of ultrasonography in predicting the development of tumor metastases in patients with breast cancer.

Upregulation of *TRIM67* was accompanied by upregulation of the pathways associated with breast cancer. In addition, the upregulation of *TRIM67* may be associated with the downregulation of certain miRNA-regulated functional pathways, including the role of membrane receptors targeted by miRNAs and response in the DNA damage response. Alterations in these pathways may be associated with the development of BM in patients with breast cancer and ultimately affect their OS. In the future, further in-depth studies are warranted to address the relationship between the role of US and related molecules in breast cancer.

There are some limitations to this study. Patients with BM from breast cancer were not compared to patients without BM as a separate group in the clinical study population. We aim to expand the sample size for further detailed analysis in the future. Additionally, we will further investigate the mechanism of *TRIM67* in breast cancer metastasis at the basic experimental level.

## Conclusion

Several risk factors were examined in this study associated with BRCA brain metastases. Ultrasound and its prediction of *TRIM67* play an important predictive role in neurovascular-related metastases from breast cancer. The nomogram of six variables—stage, *TRIM67*, tumor size, N, age, and HER2 status—were biomarkers of the likelihood of breast cancer metastasis.

## Data Availability Statement

The original contributions presented in the study are included in the article/supplementary material, further inquiries can be directed to the corresponding authors.

## Ethics Statement

The studies involving human participants were reviewed and approved by the Ethics Committee of Cangzhou Central Hospital. The patients/participants provided their written informed consent to participate in this study.

## Author Contributions

ZX, TM, YQ, and YG performed the data curation and analysis. ZX and TM analyzed and interpreted the results. ZX drafted and reviewed the manuscript. TM provided funding. All authors read and approved the final manuscript.

## Conflict of Interest

The authors declare that the research was conducted in the absence of any commercial or financial relationships that could be construed as a potential conflict of interest.

## Publisher's Note

All claims expressed in this article are solely those of the authors and do not necessarily represent those of their affiliated organizations, or those of the publisher, the editors and the reviewers. Any product that may be evaluated in this article, or claim that may be made by its manufacturer, is not guaranteed or endorsed by the publisher.

## Abbreviations

TCGA: The Cancer Genome Atlas
BRCA: Breast invasive carcinoma
BM: Brain metastasis
nDM–BRCA: BRCA with no distance metastasis
nBM–BRCA: BRCA with non-brain metastasis
TPM: Transcripts per million
DE–mRNAs: Differentially expressed mRNAs
DEGs: Differentially expressed genes
GSEA: Gene Set Enrichment Analysis
KM: Kruskal–Wallis
OS: Overall survival
qRT-PCR: Quantitative Reverse Transcription-PCR.
